# Evolution of nectarivory in phyllostomid bats (Phyllostomidae Gray, 1825, Chiroptera: Mammalia)

**DOI:** 10.1186/1471-2148-10-165

**Published:** 2010-06-04

**Authors:** Thomas Datzmann, Otto von Helversen, Frieder Mayer

**Affiliations:** 1 Museum für Naturkunde, Leibniz Institute for Research on Evolution and Biodiversity at the Humboldt University Berlin, Invalidenstr. 43, 10115 Berlin, Germany; 2 Department of Zoology, Animal Physiology, University of Erlangen-Nürnberg, Staudtstrasse 5, Erlangen, Germany; 3 Senckenberg Natural History Collections Dresden, Museum of Zoology, Königsbrücker Landstrasse 159, 01109 Dresden, Germany

## Abstract

**Background:**

Bats of the family Phyllostomidae show a unique diversity in feeding specializations. This taxon includes species that are highly specialized on insects, blood, small vertebrates, fruits or nectar, and pollen. Feeding specialization is accompanied by morphological, physiological and behavioural adaptations. Several attempts were made to resolve the phylogenetic relationships within this family in order to reconstruct the evolutionary transitions accompanied by nutritional specialization. Nevertheless, the evolution of nectarivory remained equivocal.

**Results:**

Phylogenetic reconstructions, based on a concatenated nuclear-and mitochondrial data set, revealed a paraphyletic relationship of nectarivorous phyllostomid bats. Our phylogenetic reconstructions indicate that the nectarivorous genera *Lonchophylla *and *Lionycteris *are closer related to mainly frugivorous phyllostomids of the subfamilies Rhinophyllinae, Stenodermatinae, Carolliinae, and the insectivorous Glyphonycterinae rather than to nectarivorous bats of the Glossophaginae. This suggests an independent origin of morphological adaptations to a nectarivorous lifestyle within Lonchophyllinae and Glossophaginae. Molecular clock analysis revealed a relatively short time frame of about ten million years for the divergence of subfamilies.

**Conclusions:**

Our study provides strong support for diphyly of nectarivorous phyllostomids. This is remarkable, since their morphological adaptations to nutrition, like elongated rostrums and tongues, reduced teeth and the ability to use hovering flight while ingestion, closely resemble each other. However, more precise examinations of their tongues (e.g. type and structure of papillae and muscular innervation) revealed levels of difference in line with an independent evolution of nectarivory in these bats.

## Background

The diversity of feeding specialization of phyllostomid bats are unique among all mammals [1-7]. They range from insect-to diverse vegetable-feeding strategies, as well as omnivory, carnivory, and even blood-feeding [8-16]. This ecological diversification is accompanied by morphological, behavioural and physiological adaptations [4,9,17-32]. A striking example is specialization for nectarivory, with several species feeding primarily on nectar. These bats have the ability to hover in front of a plant, while drinking nectar with their elongated and extensile tongues adorned with brush-like papillae and grooves for ingestion of nectar [3,26,29,30,33-37]. They digest and metabolize nectar and pollen quickly [32,38-44]. Phyllostomid bats represent the second largest chiropteran family after the vesper bats (Vespertilionidae Gray, 1821), with more than 150 species in at least 49 genera. Their distribution ranges from southern Arizona and the West Indies to northern Argentina [45].

Although phylogenetic analyses of morphology, chromosomes, and molecules have helped to illuminate relationships among many genera and subfamilies of phyllostomid bats, relationships among nectarivorous genera are still unclear. Many phylogenies based on morphological characters suggest a monophyletic origin for all specialized nectarivorous phyllostomids [5,46,47]. We provide a well-supported phylogenetic estimate of phyllostomid bats based on a large molecular data set, comprising 10396 bp from a total of twelve nuclear-and mitochondrial genes, and try to clarify phylogenetic relationships among nectarivorous taxa by testing whether they share a close common ancestry. Furthermore, we used a molecular clock approach to evaluate the evolutionary time frame of diversification in phyllostomid bats.

## Results

### Phylogeny of the Phyllostomidae

**Figure 1:**
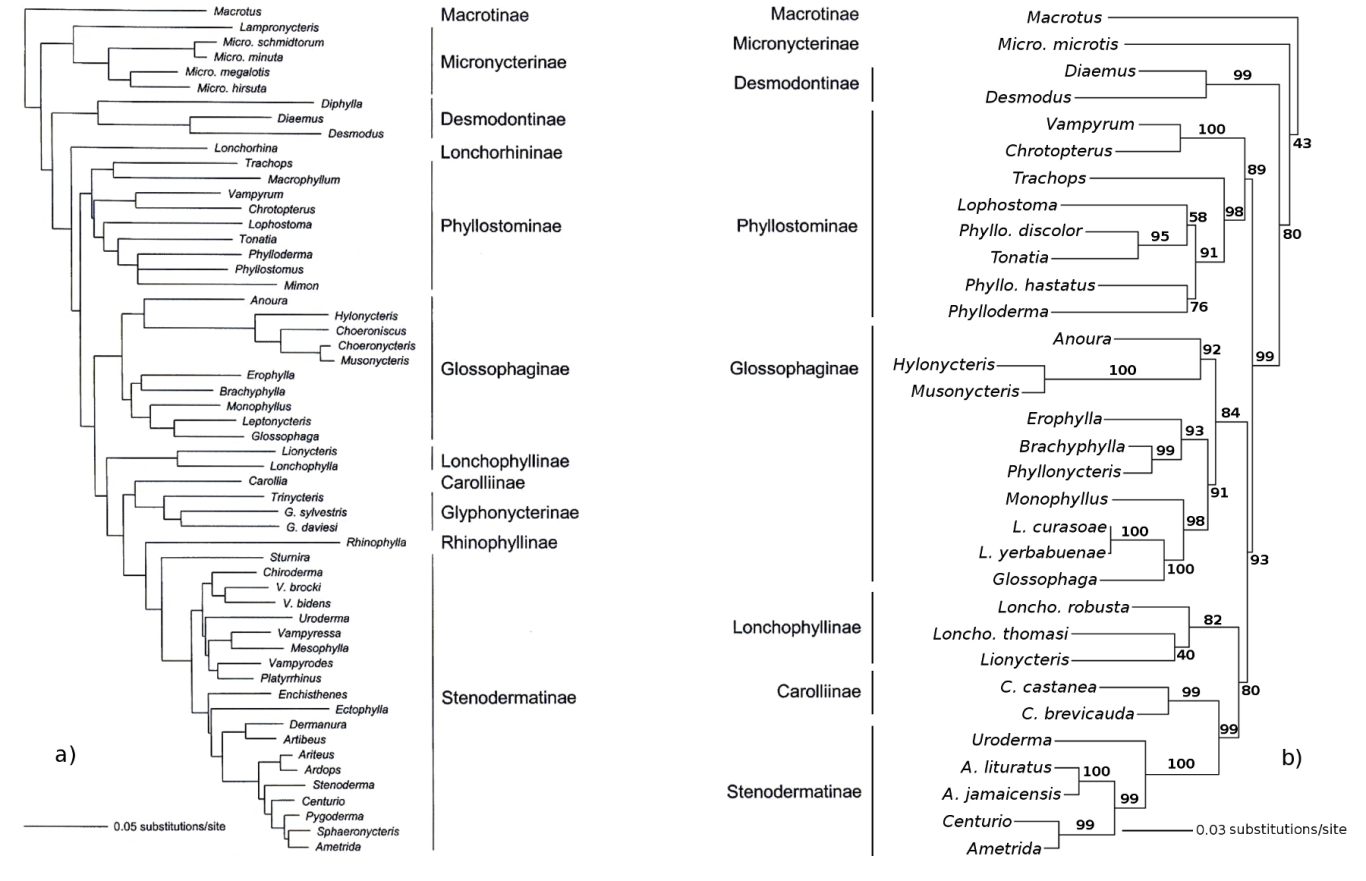
**Comparison of the phylogenies after Baker and colleagues (2003) and this paper**. a) Molecular phylogeny of phyllostomid bats after Baker *et al*. (2003) [48] based on sequences of 12S and 16S rRNA, tRNA Valin and the *rag2 *gene. b) Our molecular phylogeny inferred from a complete independent molecular data set (see methods section: Alignment 1). The taxa Lonchorhinae, Glyphonycterinae and Rhinophyllinae are missing in our reconstruction. Support values were obtained by a rapid bootstrap inference in RAxML with 500 iterations. Both phylogenies show high congruence.

Figure 1 shows our maximum-likelihood reconstruction (b) compared to the latest comprehensive analysis of phyllostomid phylogeny (a) after Baker *et al*. (2003) [48]. Baker and colleagues used sequences of 12S and 16S rRNA, tRNA Valin and the *rag2* gene for their inference. Our reconstruction shows high congruence, even though it is completely based on independent genes (see methods section: Alignment 1). Although no members of the subfamilies Lonchorhinae, Glyphonycterinae and Rhinophyllinae were included (because of incomplete data for these taxa), major branching patterns were consistently reconstructed. Our reconstruction received good bootstrap support and is in line with Baker *et al*.'s phylogeny of phyllostomid bats. Therefore, we combined our data with the data from Baker *et al*. (2003) [48].

**Figure 2:**
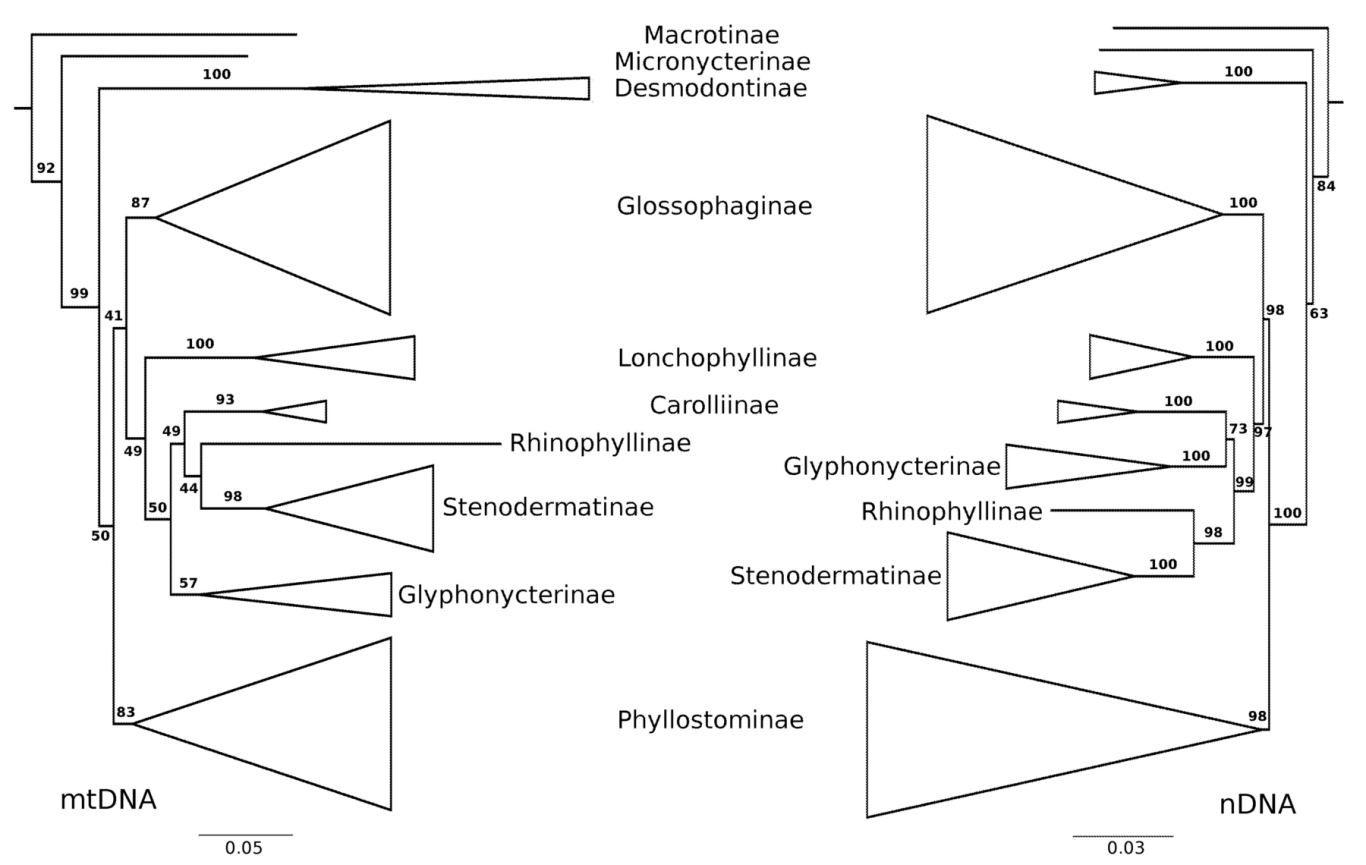
**Separate analyses of mitochondrial-and nuclear loci (see methods section: Alignment 2&3)**. Maximum-likelihood reconstruction of phyllostomid phylogeny based on concatenated mitochondrial-(left) or nuclear-(right) data. Support values were obtained by rapid bootstrap inferences in RAxML with 500 iterations. Phylogenetic relationships among different subfamilies are compared. Branches within each subfamily were collapsed. The sister group relationship between Glyphonycterinae and Carolliinae could not be supported in the reconstruction based on mitochondrial data. All other relationships are identical and all subfamilies are monophyletic in both independent reconstructions.

A separate analysis of all mitochondrial and nuclear loci (Alignment 2&3) resulted in high congruent phylogenies (Figure 2). Among the frugivorous species relationships changed between the independent inferences. A sister-group relationship between Carolliinae and Glyphonycterinae could not be inferred from the mitochondrial data set. In this reconstruction glyphonycterids were found basal to all frugivores. But this relationship obtained low support (BS 50) compared to the reconstruction based on nuclear loci, where Carolliinae is sister taxon to them (BS 73).

**Figure 3:**
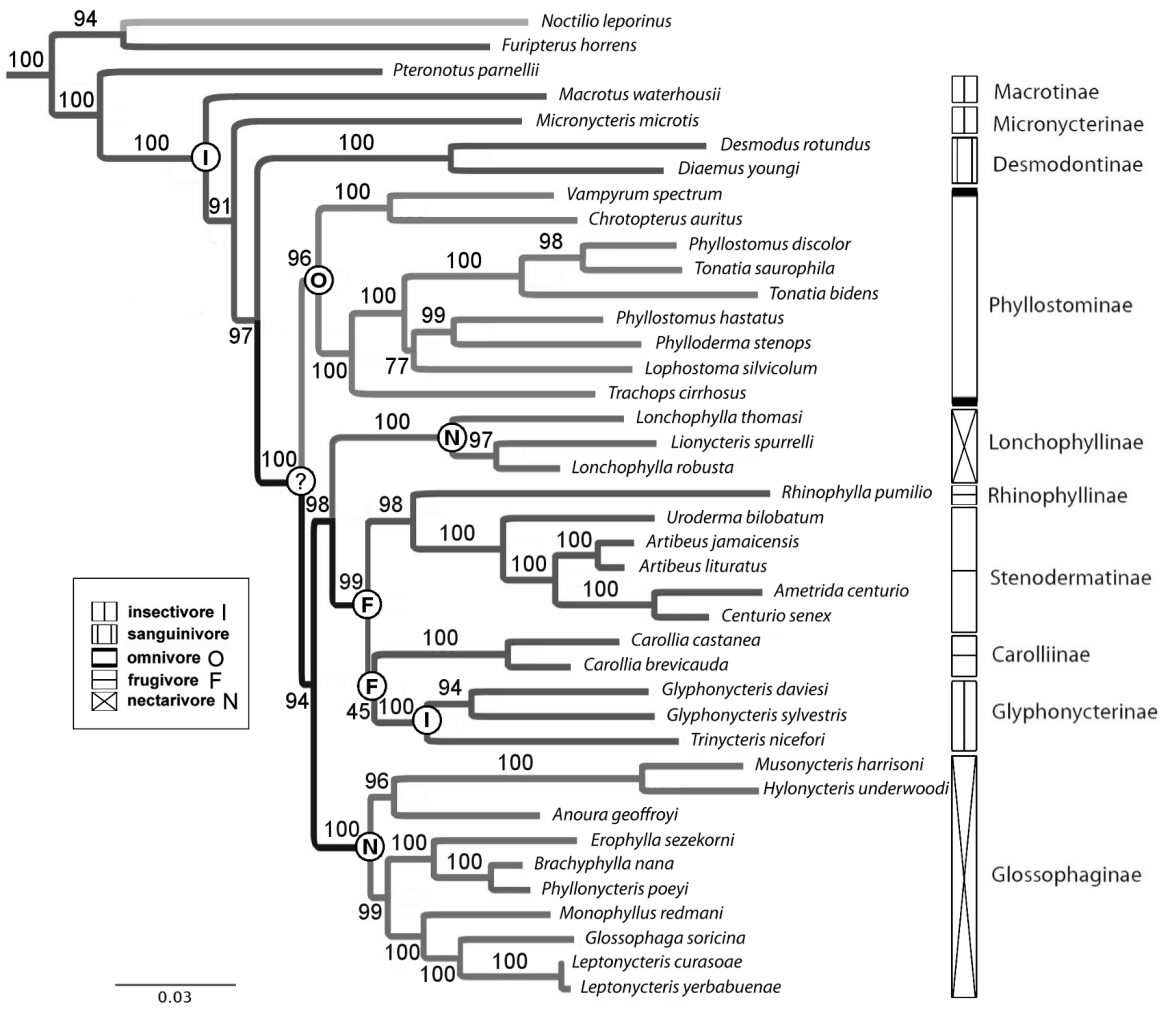
**Maximum-likelihood estimate of phyllostomid phylogeny**. Best maximum-likelihood tree obtained with RAxML v7.0.4 (see methods section: Alignment 5). The family Phyllostomidae (ten subfamilies) and representatives of closely related chiropteran families Noctilionidae, Furipteridae and Mormoopidae are shown. Further outgroup taxa (Molossidae, Vespertilionidae, Rhinolophidae) are not shown. Support values were obtained by a full non-parametric bootstrap search with 2500 iterations. Symbols refer to feeding specialization of different terminal lineages [5,61,115,116] and circles at some nodes indicate reconstructed states for their ancestors. One reconstruction obtained an ambiguous result (marked with ?).

Maximum likelihood (ML) analysis based on our supermatrix (see methods section: Alignment 5) revealed a well-resolved phylogeny for the Phyllostomidae (Figure 3), with most nodes receiving high bootstrap support (BS > 90). Monophyly of all subfamilies recognized by Baker *et al*. (2003) [48] was verified, and relevant nodes were highly supported by different measurements (Table 1).

**Table 1:** Clade stability measures.

Subfamilies Phyllostomidae	posterior	BS	DI	GMYC	branch length	congruence index
Macrotinae	n.a.	n.a.	n.a.	n.a.	0.086	1.0
Micronycterinae	n.a.	n.a.	n.a.	n.a.	0.073	1.0
Desmodontinae	1	100	64	yes	0.049	1.0
Phyllostominae	1	99	3	yes	0.004	0.6
Lonchophyllinae	1	100	27	yes	0.031	1.0
Rhinophyllinae	n.a.	n.a.	n.a.	n.a.	0.090	1.0
Stenodermatinae	1	100	28	no	0.023	0.6
Carolliinae	1	100	25	yes	0.034	1.0
Glyphonycterinae	1	100	8	yes	0.013	0.6
Glossophaginae	1	100	36	no	0.014	0.6

Different measures were calculated to describe clade stability: posterior probability, bootstrap value (BS), decay index (DI), Bayesian cluster recognition (GMYC) and branch length. Further, trophic level of the bats was also considered (Figure 3). Decay indices were obtained with TreeRot.v3 [117] in combination with the phylogenetic software PAUP 4.0 beta [89]. A decay index greater than ten is considered as strong support for a specific node. Automated cluster recognition via the "Generalized Mixed Yule-Coalescent" (GMYC) approach with multiple threshold extension was used [118-120]. Branches longer than the mean branch length (0.03 substitutions per position) are considered as long. Congruence indices were calculated by the number of useable support measures for a specific node divided by the number of cases in which there was high support.

Three basal lineages, comprising the taxa Macrotus (1), Micronycteris (2), and the vampire bats Desmodus and Diaemus (3), were confirmed (Figure 3). A bifurcation in more or less omnivorous bats (Phyllostominae) and predominantly vegetarian species followed. Within the frugivores a sister-group relationship between Rhinophyllinae and the Stenodermatinae was well-supported (BS 99). However, support for a sister-group relationship of Carolliinae and Glyphonycterinae was weak (BS 48). The highly specialized nectarivorous taxa *Lonchophylla *and *Lionycteris *do not align closely with other nectarivorous phyllostomids (Glossophaginae). Instead, they shared a common ancestor with the frugivores, as previously proposed by Baker *et al*. (2003) [48] and others [33,49,50].

### Divergence time estimation and model decision

**Figure 4:**
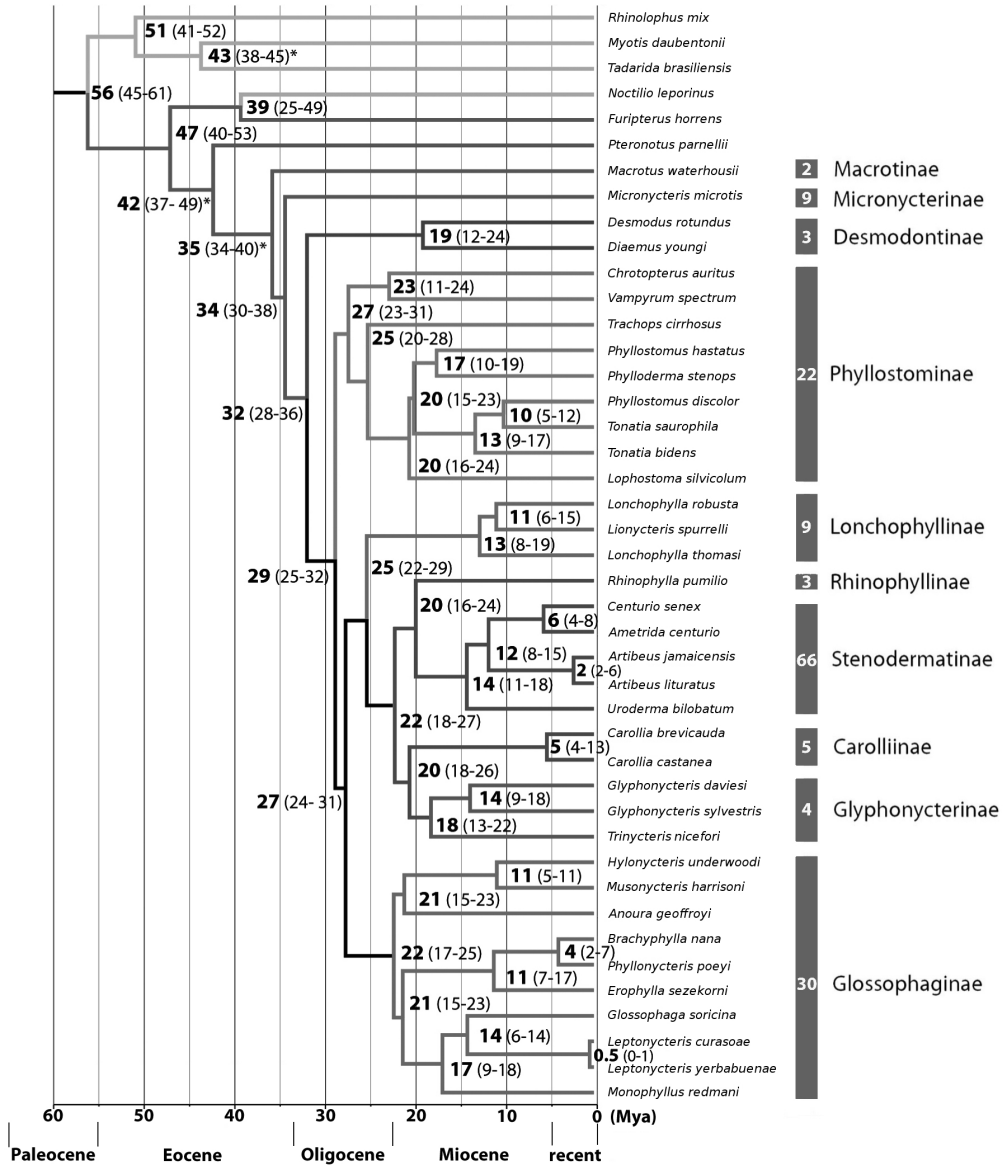
**Bayesian dating of phyllostomid diversification**. Maximum clade credibility tree under the UCLN model in BEAST built on 48.003 sampled trees. The *Geological Time Scale *(2004) of *The International Commission on Stratigraphy *(ICS) was used as a timetable. Node ages (bold) in million years ago (Mya) with their 95% HPD interval (in parenthesis) are shown, rounded to nearest integer. 95% HPD ranges can be seen as confidence intervals. Nodes marked with an asterisk are calibrated with fossils. Absolute species numbers within each subfamily, according to the actual species list [45], are given in the broad vertical bars.

The analysis under the lognormal relaxed clock model (UCLN) produced the smallest confidence intervals compared to the exponential-(UCED) or strict clock model (CLOC). Estimates of mean likelihood, substitution rate, and node age were most accurately inferred under the UCLN model (Table 2). The assumption of the relaxed clock, that branches differ in their substitution rates, was confirmed. A coefficient of variation of 0.405 indicated moderate rate variation [51]. Figure 4 shows the dated Bayesian tree inferred with BEAST under the UCLN model. The common ancestor of all phyllostomids was dated to the Middle Eocene (42 MYA), with a confidence interval between 49- and 37 MYA. Basal lineages within the phyllostomids arose shortly thereafter in the Late Eocene or Early Oligocene (35-32 MYA). The prominent amount of the remaining lineages emerged in a time frame of about ten million years at the transition from Oligocene to Miocene (29-20 MYA), with 21 out of 33 lineages already present in the Early Miocene (20 MYA).

**Table 2:** Model comparison.

Molecular Clock Model	CLOC - 3 priors			CLOC (without data)		
	mean	< 95% HPD	> 95% HPD	mean	< 95% HPD	> 95% HPD
Likelihood	-8.42E+004	-8.42E+004	-8.42E+004	-	-	-
rate [**]	1.40E-003	1.28E-003	1.52E-003	50.3	4.82	99.61
rootHeight [*]	61.04	56.42	66.05	60.75	40.81	86.73
Molossidae × Vespertilionidae p1 [*]	39.32	37.49	41.64	41.15	37.64	46.01
Mormoopidae × Phyllostomidae p2 [*]	56.4	51.3	61.86	38.39	34.09	44.75
Macrotus × rest of Phyllostomidae p3 [*]	47.99	43.64	52.57	35.71	34	39.1

Molecular Clock Model	UCED - 3 priors			UCLN - prior p1		
	**mean**	**< 95% HPD**	**> 95% HPD**	**mean**	**< 95% HPD**	**> 95% HPD**

Likelihood	-7.68E+004	-7.69E+004	-7.68E+004	-7.68E+004	-7.68E+004	-7.68E+004
rate [**]	3.96E-003	3.04E-003	4.81E-003	1.90E-003	1.15E-003	2.73E-003
rootHeight [*]	62.9	42.24	90.14	81.84	51.92	116.2
Molossidae × Vespertilionidae p1 [*]	41.5	37.71	46.88	42.43	37.71	49.8
Mormoopidae × Phyllostomidae p2 [*]	39.24	34.77	46.91	77.49	54.67	104.63
Macrotus × rest of Phyllostomidae p3 [*]	35.49	34	38.5	67.49	37.86	100.44

Molecular Clock Model	UCLN - prior p2			UCLN - prior p3		
	**mean**	**< 95% HPD**	**> 95% HPD**	**mean**	**< 95% HPD**	**> 95% HPD**

Likelihood	-7.68E+004	-7.68E+004	-7.68E+004	-7.68E+004	-7.68E+004	-7.68E+004
rate [**]	4.09E-003	3.09E-003	4.95E-003	3.50E-003	2.94E-003	4.07E-003
rootHeight [*]	38.32	30.94	49.32	42.73	34.99	51.79
Molossidae × Vespertilionidae p1 [*]	22.89	12.51	34.16	22.39	12.97	33.52
Mormoopidae × Phyllostomidae p2 [*]	34.99	30.37	42.93	37.1	28.73	46.06
Macrotus × rest of Phyllostomidae p3 [*]	29.07	23.35	37.01	35.81	34	39.43

Molecular Clock Model	UCLN - 2 priors p1+p3			UCLN - 3 priors		
	**mean**	**< 95% HPD**	**> 95% HPD**	**mean**	**< 95% HPD**	**> 95% HPD**

Likelihood	-7.68E+004	-7.68E+004	-7.68E+004	-7.68E+004	-7.68E+004	-7.68E+004
rate [**]	3.04E-003	2.58E-003	3.43E-003	3.13E-003	2.76E-003	3.48E-003
rootHeight [*]	53.85	45.94	63.48	52.26	45.42	61.28
Molossidae × Vespertilionidae p1 [*]	40.99	37.7	45.85	40.89	37.64	45.39
Mormoopidae × Phyllostomidae p2 [*]	44.56	37.34	53.21	42.16	37.13	48.61
Macrotus × rest of Phyllostomidae p3 [*]	36.96	34	42.72	35.82	34	39.58

Divergence time estimations of specific nodes under different molecular clock models and different calibration settings are shown. Strict- (CLOC), relaxed exponential- (UCED) and relaxed lognormal- (UCLN) clock models are compared. Likelihood value, mean mutation rate, root age and time to the most recent common ancestor (tMRCAs) of taxon subsets are given. [*] Estimated age of taxon subset in million years ago (Mya). [**] Estimate of the evolutionary rate across the whole tree in units of substitutions per site per million years (Myr). < > Lower and upper bound of the 95% highest posterior density (HPD) interval. 95% HPD is the shortest interval, that contains 95% of the sampled values and is equivalent to a confidence interval.

### Reconstruction of ancestral states

Figure 3 shows the reconstruction of ancestral states by the maximum-likelihood approach under the Markov k-state model. Only relevant nodes, which will be used in the discussion chapter, are shown. Reconstructed feeding specialization of the common ancestor of all phyllostomids and of the common ancestor of important clades were mapped on the tree (Figure 3). Unambiguous character states were assigned to nodes with a probability of more than 90% for one reconstructed state. The feeding specialization of the common ancestor of all omnivorous and predominantly vegetarian phyllostomid species could not be resolved, as the reconstruction was ambiguous for this node (marked with ?). We obtained probability values of 47% for a nectarivorous-, 39% for an omnivorous-, and 12% for an insectivorous state at this node.

## Discussion

### Phylogeny of the Phyllostomidae

Our molecular phylogenetic reconstructions based on more than 10 kb DNA sequences obtained high bootstrap support for almost all nodes and challenges several phylogenetic relationships derived from morphological data sets. Our results partly disagree with recent classifications of phyllostomid bats [45,49] including: (1) placement of insectivorous genera *Macrotus*, *Micronycteris*, *Glyphonycteris *and *Trinycteris *within the Phyllostomidae; (2) relationship of the fruit-eating genus *Rhinophylla *to other frugivores; and (3) relationships among nectarivorous phyllostomids.

The molecular data suggest that the genera *Macrotus *and *Micronycteris *do not belong to the subfamily Phyllostominae as proposed by Koopman (1994) [49], McKenna and Bell (1997) [47], Wetterer *et al*. (2000) [5], and Jones *et al*. (2002) [50]. Instead, they form two divergent basal lineages within phyllostomid bats (Figure 3). Our data are in line with the findings of Baker *et al*. (2003) [48]. The authors proposed a classification of two different subfamilies Macrotinae and Micronycterinae. Three studies placed the genera *Glyphonycteris *and *Trinycteris *within the subfamily Phyllostominae [5,45,49]. In contrast, our data revealed a close relationship of *Glyphonycteris *and *Trinycteris *with frugivorous species of the subfamily Carolliinae. Despite low support for this sister-group relationship (BS 45), our data support a closer relationship of *Glyphonycteris *and *Trinycteris *to fruit-eating species (BS 99) than to omnivorous phyllostomids of the subfamily Phyllostominae.

The genus *Rhinophylla *does not belong to the subfamily Carolliinae, as proposed by McKenna and Bell (1997) [47], Wetterer *et al*. (2000) [5], and Jones *et al*. (2002) [50]. Our data support a sister-group relationship between *Rhinophylla *and the subfamily Stenodermatinae, as proposed by Baker *et al*. (2003) [48].

Many authors excluded the genera *Phyllonycteris*, *Erophylla *and *Brachyphylla*, all endemic to the West Indies, from other nectarivorous phyllostomids and placed them mostly into the subfamilies Phyllonycterinae and Brachyphyllinae [3,5,19,33,45,49,50,52-59]. In contrast, our data show that these nutritionally more generalized bats belong to the Glossophaginae (BS 100). The three genera are closely related to more specialized nectarivorous bats of the genera *Glossophaga*, *Leptonycteris *and *Monophyllus*. This is in line with an earlier molecular phylogeny of Baker *et al*. (2003) [48]. The phylogenetic position of highly specialized nectarivorous bats of the genera *Lonchophylla *and *Lionycteris *is controversial. Several studies, primarily based on morphological analyses, placed them within the Glossophaginae [5,46,47,52]. Instead, our molecular data suggest that they are closer related to Rhinophyllinae, Stenodermatinae, Carolliinae, and Glyphonycterinae than to the Glossophaginae. This finding is in line with previous studies of Koopman (1994) [49], Jones *et al*. (2002) [50] and Baker *et al*. (2003) [48]. The distinctness of Lonchophyllinae is also supported by fixed differences in the tongue morphology (see below) between representatives of the Lonchophyllinae and Glossophaginae [33].

In summary, our study supports the classification of phyllostomid bats after Baker *et al*. (2003) [48]. Their division into more subfamilies, compared to Koopman (1994) [49] and Simmons (2005) [45], seems justifiable, because this better reflects the remarkable ecological diversity of this family.

### Dietary diversification

The vast majority of bats feed on insects [4]. This includes the family Mormoopidae, which represents the sister group of the Phyllostomidae. In addition, the diet of the most basal subfamilies Macrotinae and Micronycterinae consists mainly of insects (Figure 3). These findings indicate, that the common ancestor of phyllostomid bats was an insect-feeder. This supposition is also supported by the maximum-likelihood reconstruction of the ancestral state (Figure 3).

Members of the Phyllostominae have a mixed diet. The reconstruction of the ancestral state for this group revealed that their physiological pre-adaptations to omnivory could have evolved only once, and involved metabolic changes from insectivorous to an omnivorous diet. However, too little is known about the diet of these bats. A high spacial and seasonal plasticity is observed [60]. A few members of the Phyllostominae are carnivorous and feed on small vertebrates [61]. For example, Trachops cirrhosus is specialized on tungara frogs [62,63]. Such a unique specialization likely evolved in a formerly insectivorous/omnivorous species. It was shown, for the seasonally carnivorous Greater Noctule bat (*Nyctalus lasiopterus*, Vespertilionidae), that only minor changes are needed to switch from insectivory to carnivory (inclusion of small vertebrates in the diet) [64,65]. The transition from large-bodied insects to small vertebrates as prey does not need any major adaptations and occurred several times independently in different bats and is correlated with an increase in body size [61].

A large number of phyllostomid species have a vegetarian diet. They form a monophyletic clade (BS 99), comprising the subfamilies Rhinophyllinae, Stenodermatinae, Carolliinae, Glossophaginae, Lonchophyllinae, and surprisingly the Glyphonycterinae. The last subfamily includes several strict insectivorous species; thus, a shift from a vegetarian diet back to insectivory seems to be the most plausible scenario. Alternatively, the Glyphonycterinae retained the ancestral insectivorous lifestyle. This assumption would require that the frugivorous subfamilies Rhinophyllinae, Stenodermatinae and Carolliinae have evolved their feeding specialization independently from each other. However, the relevant node is weakly supported in our phylogenetic reconstruction. It is also possible (see Figure 2 based on mitochondrial data) that the Glyphonycterinae represents a basal lineage to all frugivores and therefore possess the plesiomorphic state for this group. The common ancestry of all frugivore species was also postulated by previous studies [5,46,48,50,59]. However, there is a high dietary plasticity in this group. For example, *Carollia *is a known switch hitter between fruits and insects depending on the season (summarized in [66]).

The diphyly of the nectarivorous Lonchophyllinae and Glossophaginae is surprising, since they resemble each other in many morphological, behavioural, ecological, and physiological traits (e.g. skull elongation, reduction of dentition, hovering flight, forest foraging behaviour and ability to metabolize pollen). Accordingly, these similarities have evolved independently by natural selection during the adaptation to a nectar-feeding lifestyle. This hypothesis is supported by some obvious differences in these adaptive traits [33]: The lonchophyllines have a deep longitudinal groove in their tongue, lined dorsal and ventral with hairlike papillae. This groove is missing in the glossophagines and hairlike papillae are distributed anterodorsal, forming a brush tip. Furthermore, the lonchophyllines lost most types of papillae found on the tongues of other phyllostmids, including the glossophagines. Also, the internal tongue structure is very different. The lonchophyllines have complex, omnidirectional bundles of muscles within the tongue, while glossophagines have predominantly horizontal skeletal muscle bundles. The complex orientated muscles in the lonchophyllines are supposed to control the shape of the groove during nectar feeding [33]. Drinking behaviour varies widely between both subfamilies (Marco Tschapka, pers. comm., [30]). Other characters show similar apomorphic states in lonchophyllines and some glossophagines (e.g. posterior shift of sternohyoid origin, xiphoid origin of sternohyoid, elongated hyoglossus and loss of connection to hyoid bone, double insertion of geniohyoid, posterior shift of genioglossus insertion [33]), however, there are no consistent patterns. The endemic West Indian genera, *Brachyphylla*, *Erophylla*, and *Phyllonycteris*, show many plesiomorphic characters. It seems that functional constraints on the muscular innervation of the tongue curtain the evolutionary signals of these characters. Hence, it is possible that lonchophyllines and glossophagines may have evolved these adaptations for nectar-feeding independently (but see also [67,68]).

The large number of species within the clade of frugivorous and nectarivorous bats (Figure 2) suggests, that a shift to a vegetarian diet accelerated the diversification rates in this group. The majority of phyllostomid bats, 117 out of 158 listed species [45], i.e. 74%, belong to this clade. Possibly the presence of numerous vacant ecological niches in tropical and subtropical regions of America (see also [69]) resulted in allopatric speciation.

### Time frame of evolution

Our analysis revealed a time frame of ten million years (29-20 MYA) from Oligocene to Early Miocene, in which all prominent lineages evolved (Figure 4). Most of the species diversification occurred subsequent to the Oligocene epoch (since 23 MYA). During the Miocene substantial changes of the landscape occurred in Tropical America due to massive plate tectonics [70-75]. Global climate cooled and resulted in an increase in aridity [76-78]. Frequent isolation events could have resulted in allopatric populations and thus promoted speciation [79]. Interestingly, the radiation of extant hummingbirds (Trochilidae), another alimentary competitor, shows a similar pattern of diversification in the Middle Miocene [80]. Geologic upheavals as well as the ability of ecologically generalized species to invade new regions were considered as major forces promoting hummingbird radiations in newly arisen montane regions. In order to test whether these factors has also promoted speciation in bats, and to infer other underlying evolutionary mechanisms, a much denser taxon sampling is required.

## Conclusions

Our analysis of more than 10.000 base pairs of concatenated DNA sequences reveals a strongly supported phyllostomid phylogeny, thus allowing for clear predictions about the evolution of feeding specialization of these bats. Several morphological and even molecular studies were unable to resolve the specific branches with sufficient support, either due to the convergent nature of the analyzed characters or insufficient amount of sequence data. Our multi-gene approach, combined with a relaxed clock analysis, detected and dated major splitting events within this family. This study gives support for the classification of phyllostomid bats after Baker *et al*. (2003) [48]. All prominent lineages with diverse feeding strategies evolved within a relatively short time frame of about ten million years from Oligocene to Early Miocene. Geological and climate changes as well as the shift to a vegetarian diet may have promoted the radiation into diverse lineages. In this context, the diphyly of the nectarivorous Lonchophyllinae and Glossophaginae is remarkable. Despite many similarities between both groups, it seems plausible, that they evolved their adaptations to nectarivory independently from each other. This would represent an example of convergent evolution within bats that led to very similar features, which play a major role in food acquisition.

## Methods

### Taxon sampling

Thirty-seven phyllostomid species of 29 genera were analyzed. Our sampling comprises members of all extant subfamilies [45,49], except bats of the subfamily Lonchorhinae. We used species and subfamily assignments according to Baker *et al*. (2003) [48]. One representative each of the families Mormoopidae, Furipteridae, Noctilionidae, Molossidae and Vespertilionidae were used as outgroup taxa. Two closely related specimens were used for the family Rhinolophidae, because we were not able to analyze all loci entirely for one taxon. GenBank accession numbers are given in addtitional file 1. Tissue samples were provided by cooperation partners (see acknowledgements). The name of the body which gave approval and corresponding reference numbers could be obtained from them.

### Genetic analyses

Extraction of total genomic DNA was done by Chloroform-Isoamyl-Phenol precipitation. A 1.3 kb fragment of the exon 28 of the von Willebrand factor gene (*vwf*) was amplified with the primers vWF-A and vWF-B [81], or with vWF-A and vWF-B2 [81] within a Nested PCR. Primer vWF-B2 anneals 139 bp upstream from vWF-B. An approximately 1.4 kb fragment of the recombination activating gene 2 (*rag2*) was amplified with the primers RAG2-F1 and RAG2-R2 [59], or with RAG2-F1B and RAG2-R2 [59]. The PCR Mastermix (25 *μl *final reaction volume) included 2 *μl *of total genomic DNA extract, 1.25 *μl *of each primer (10 *μM*), 1 *μl *of MgCl_2_ (25 *mM*), 1 *μl *of a dNTP-Mix (10 *mM*) and 1 unit of Peglab Taq polymerase. Nested PCR was performed using 2 *μl *from a 1:40 delution of the first PCR reaction. The fragments were amplified following a Two-Step protocol. Thermocycling consisted of a 3 min initial denaturation at 95°C, followed by 5 cycles of 30s at 95°C, 50s at 65°C (for the *vwf*), or 30s at 60°C (for the *rag2*), and 90s at 72°C. 35 cycles with 50s annealing at 62°C (for the *vwf*) and 30s at 57°C (for the *rag2*) were performed, followed by a final extension of 6 min at 72°C. A fragment of exon 11 of the breast cancer susceptibility gene (*brca1*) was amplified with the primers BRCA1-F126 [82] and a newly designed (ER 515: 5'- AAGTGTTGGAAGCAGGGAAGCTCTTC-3'). The PCR-Mastermix (50 *μl *final reaction volume) included 2 *μl *of total DNA extract, 2.5 *μl *of each primer (10 *μM*) and 25 *μl *Phusion Mastermix. Thermocycling consisted of a 30s initial denaturation at 98°C, followed by 5 cycles of 10s of 98°C, 25s at 66°C, and 90s at 72°C. 30 cycles with 25s annealing at 63°C were performed, followed by a final extension of 6 min at 72°C. Two non-coding nuclear loci were also analyzed: 3'-UTR region of the phospholipase C beta 4 gene (*plcb4*) [83] and short intron of the phosphoenolpyruvate carboxykinase gene (*pepck*) [84];

We amplified a mitochondrial fragment of the NADH dehydrogenase subunit 1 gene (*nd1*) and the tRNA Leucin, using the primers ER 65 and ER 66 [85]. Published sequences of five additional mitochondrial loci (COI, Cytb, 12S rRNA, 16S rRNA and tRNA Valin) were incorporated. For all analyses, the ribosomal RNAs and the tRNA Valin were combined (12StRNA16S). Accession numbers are given in Additional file 1. It also includes an overview of all incorporated taxa, loci and sequences and the percentage of missing data per species, as well the geographic origin of our samples. The overall amount of missing data is about 30%.

### Alignments and model selection

All alignments were done with Sequencher v4.7 [86] and Bioedit v7.0.9 [87,88] and checked manually by eye. We performed bootstrap analyses of each individual loci to check for compatibility of their individual phylogenetic signal. Because none of the strongly supported clades based on individual loci were mutually incompatible, we concatenated all loci except the ribosomal RNAs, tRNA Valin and the *rag2 *gene. These loci were already used by Baker and colleagues to infer a molecular phylogeny of phyllostomid bats [48]. We avoided in a first step the inclusion of them to get an independent data set [Alignment 1]. In a second step we concatenated all mitochondrial loci (this time with the inclusion of the ribosomal RNAs and the tRNA Valin) [Alignment 2] and also all nuclear loci (with *rag2*) [Alignment 3]. We concatenated all loci into one supermatrix for the final analyses. The supermatrix contained three nuclear protein-coding genes (*rag2*, *vwf *and *brca1*), two non-coding nuclear markers (*pepck*, *plcb4*), three mitochondrial protein-coding genes (*co1*, *cytb *and *nd1*), two tRNAs (Valin, Leucin) and two mitochondrial rRNAs (12S, 16S). For the Bayesian analyses, we excluded all 3rd codon positions in the mitochondrial protein-coding genes because they showed a high degree of homoplasy (homoplasy index, HI = 0.75 - parsimony analysis of the 3rd codon positions in PAUP 4.0 beta [89]). Such high homoplastic characters give a misleading phylogenetic signal and lead especially to an underestimation of real branch lengths. Therefore, we excluded them from the analyses. This resulted in a final length of 10396bp, including 2761 parsimony informative characters [Alignment 4 - Additional file 2]. For the maximum-likelihood analyses, we used a second alignment, in which the mitochondrial protein-coding sequences were translated in amino acids and combined with the remaining DNA sequences [Alignment 5 - Additional file 3]. The best fitting evolutionary model for the protein data was inferred with Prottest v1.4 [90]. The MTMAM model, designed for the evolution of mitochondrial proteins of mammals [91], showed the highest fit. We ran jModelTest [92] for the remaining DNA sequences separate for the alignments 1-5. Except for alignment 3, GTR+Γ [93] was proposed to be the best fitting evolutionary model according to Akaike- (AIC) and Bayesian (BIC) information criterion [94,95]. The slightly simpler Symmetrical Model SYM+Γ [96] was proposed for alignment 3 by jModelTest. However, we also used the GTR+Γ model for this data set for general compatibility among the inferences. Genes could have a different sequence evolution. Therefore, we generated five partitioning schemes [97] for alignment 5 to decide, which is the best adjustment for our analysis: (1) no partitioning; (2) mitochondrial- and nuclear loci separately; (3) three partitions; (4) eight partitions; and (5) 14 partitions with partitioning into codon positions for all nuclear genes. According to AIC and BIC, scheme 5 was preferred.

### Maximum-Parsimony analysis

Equal weighted maximum-parsimony (MP) analyses were performed with PAUP 4.0 beta [89] with a heuristic search using the TBR (tree-bisection-reconnection) algorithm for branch swapping. Bootstrap inferences were conducted separately for each loci with 500 pseudoreplicates.

### Maximum-Likelihood analysis

Maximum-likelihood (ML) inferences were performed with RAxML v7.0.4 [98-100]. ML searches were conducted with the rapid hill-climbing algorithm [101,102] under GTR+Γ with four rate categories as model of evolution. Multiple independent runs were started to get an impression of the robustness of the phylogenetic reconstruction. Support values were obtained through a full non-parametric bootstrap- or rapid bootstrap inference (stated for each analysis).

### Reconstruction of ancestral states

Ancestral character states were reconstructed in Mesquite v2.71 [103]. Observed character states (insectivore, sanguinivore, omnivore, frugivore and nectarivore) of the main diet were mapped on the original maximum-likelihood tree (Figure 3). We used the "Trace Character History" analysis with a symmetric, one-parameter Markov k-state model [104,105], which computes likelihoods for categorical characters, and reconstructs ancestral states by the maximum marginal probability (MLE) criterion.

### Bayesian analysis

Bayesian inferences were performed with BEAST v1.4.8 [106]. The searches were conducted under Hasegawa-Kishino-Yano HKY+Γ [107] with four rate categories as model of evolution. We chose a simpler model of sequence evolution for the Bayesian analyses as proposed by jModelTest because there was a trade-off between computational power and model complexity. It was not possible to get a consistent phylogenetic reconstruction between different runs under the GTR+Γ model in reasonable time. Bayes factor analysis between these runs under the GTR+Γ model resulted always in values far above 20 and sampling efficiency was drastically reduced compared to the HKY+Γ model. A high Bayes factor is a sign for incompatibility and poor convergence among the trees gathered in independent runs.

### Calibration of the molecular clock

We incorporated three different calibration points including: (1) divergence between Vespertilionidae and Molossidae set at 37 million years ago (MYA) in the Middle Eocene [47]; (2) age of the Mormoopidae oldest fossils from Whitneyan (30-32 MYA) land deposits in Florida [108]; and (3) age of the oldest crown group fossils of the phyllostomids in the Laventan about 11.8 to 13.8 MYA [109] and age of the oldest stem group fossils in the Whitneyan within the Early Oligocene [110]. We used the proposed age of the fossils and lognormal distributions to model minimum age constraints for the specific nodes (1,2). Maxmimum age constraints were set to the Cretaceous-Tertiary boundary at 65 MYA (1,2). Additional, a maximum age constraint for the phyllostomids (3) was set with an exponential distribution to 34 MYA with an arbitrarily lower limit of 11.5 MYA.

### Model- and prior decision

We performed several Bayesian inferences under one strict (CLOC) and two relaxed (UCLN, UCED) molecular clock models [111,112]. Always 10 million steps were performed. We examined the joint influence of the calibrations on the divergence time estimates by running a strict clock model with fixed topology, but with no sequence data. Further, we examined the influence of each individual calibration by running several inferences under an uncorrelated lognormal relaxed clock model (UCLN) with all possible combinations of the three calibrations. A precise examination and comparison of the results were performed in Tracer v1.4 [113]. An overview of important parameters for model comparison is given in Table 2. Important parameters, such as mean likelihood value, substitution rate, and node age, were calculated for every inference and compared with each other. Confidence intervals measured as 95% highest posterior density interval (HPD) were also computed. The clock model that produced the smallest confidence intervals altogether was considered most appropriate for the data [112].

### Estimation of divergence times

We conducted three independent runs for the final divergence time estimates under the UCLN model with 20 million inferences and a sample frequency of 1000 steps. We used always the same parsimony tree as starting point. We compared the results and calculated pairwise Bayes factors for the difference in their marginal likelihoods. The first 4 million steps were cut off as burnin for each comparison. Low Bayes factors are a sign for high convergence of the values and compatibility of the inferences, while high Bayes factors indicate incompatibility. Individual runs were combined with LogCombiner, TreeAnnotator and analyzed with Tracer v1.4 and FigTree v1.1.2 [114]. TreeAnnotator and LogCombiner are provided as part of the BEAST package.

## Authors' contributions

OvH was the initiator of this study. FM supervised the whole project, gave many ideas, helped to evaluate the results and to draw up the manuscript. TD made all the lab work, performed the phylogenetic analyses and wrote the manuscript.

## Supplementary Material

Additional file 1 **Incorporated sequences**. GenBank accession numbers of all incorporated sequences are shown. Dotted lines indicate missing data. Percentage of overall missing base pairs per lineage are given (completeness). Sample origins of our analyzed individuals are coded with two-letter abbreviations according to the *International Organization of Standardization*: RU Russia, CU Cuba, CR Costa Rica, JM Jamaica, MX Mexico, p.e. GenBank sequences published earlier. Question marks (?) are used for samples with unknown origin. [*] Asterisks indicate sequences published within this paper and were submitted to EMBL-EBI database hosted by the *European Molecular Biology Laboratory*. Click here for file

Additional file 2 **Input file for the Bayesian analysis in BEAST (see methods section: Alignment 4)**. XML formatted input file for the Bayesian analysis in BEAST. Can be opened within a browser, or executed with the software BEAST. Click here for file

Additional file 3 **Concatenated alignment for the maximum-likelihood inference with RAxML (see methods section: Alignment 5)**. Alignment file is in PHYLIP format and can be viewed with every text editor, or used directly in most phylogenetic software packages. Click here for file
